# Lipid Nanomaterials for Targeted Delivery of Dermocosmetic Ingredients: Advances in Photoprotection and Skin Anti-Aging

**DOI:** 10.3390/nano12030377

**Published:** 2022-01-24

**Authors:** Eliana B. Souto, Eliézer Jäger, Alessandro Jäger, Petr Štěpánek, Amanda Cano, Cesar Viseras, Raquel de Melo Barbosa, Marlus Chorilli, Aleksandra Zielińska, Patricia Severino, Beatriz C. Naveros

**Affiliations:** 1 CEB—Centre of Biological Engineering, Campus de Gualtar, University of Minho, 4710-057 Braga, Portugal; 2 LABBELS—Associate Laboratory, Braga, 4800-122 Guimarães, Portugal; 3 Macromolecular Chemistry Institute, Academy of Sciences of the Czech Republic, Heyrovského nº. 2, 162 06 Prague, Czech Republic; eliezerjager@hotmail.com (E.J.); alejager@gmail.com (A.J.); stepanek.p@vutbr.cz (P.Š.); 4 Department of Pharmacy, Pharmaceutical Technology and Physical Chemistry, Faculty of Pharmacy and Food Sciences, University of Barcelona, 08028 Barcelona, Spain; acanofernandez@ub.edu; 5 Institute of Nanoscience and Nanotechnology (IN2UB), University of Barcelona, 08028 Barcelona, Spain; 6 Department of Pharmacy and Pharmaceutical Technology, School of Pharmacy, Campus of Cartuja s/n, University of Granada, 18071 Granada, Spain; cviseras@ugr.es (C.V.); patricia_severino@itp.org.br (P.S.); 7 Andalusian Institute of Earth Sciences, CSIC-University of Granada, Av. de Las Palmeras 4, Armilla, 18100 Granada, Spain; m.g.barbosafernandes@gmail.com; 8 Laboratory of Drug Development, Department of Pharmacy, School of Pharmacy, Federal University of Rio Grande do Norte, Natal 59078-970, Brazil; 9 Faculty of Pharmaceutical Sciences, Campus of Araraquara, São Paulo State University (UNESP), Rodovia Araraquara Jaú, Araraquara 14800-903, Brazil; marlus.chorilli@unesp.br; 10 Institute of Human Genetics, Polish Academy of Sciences, Strzeszyńska 32, 60-479 Poznań, Poland; aleksandra.zielinska@igcz.poznan.pl; 11 Institute of Technology and Research (ITP), University of Tiradentes (UNIT), Av. Murilo Dantas, 300, Aracaju 49010-390, Brazil; 12 Industrial Biotechnology Program, University of Tiradentes (UNIT), Av. Murilo Dantas, 300, Aracaju 49010-390, Brazil; 13 Biosanitary Institute of Granada (ibs.GRANADA), 18012 Granada, Spain

**Keywords:** sun exposure, solar photoprotection, lipid nanofilms, ultraviolet radiation

## Abstract

Despite the health benefits of the sun, overexposure to solar radiation without proper precautions can cause irreversible damage to exposed skin. In the search for balance between the risks and benefits of exposure to solar radiation in human health, a technological alternative was found, the incorporation of photoprotective products in lipid nanoparticulate systems for topical application. These nanometric systems have demonstrated several advantages when used as adjuvants in photoprotection compared to chemical and/or physical sunscreens alone. The increase in the sun protection factor (SPF), photostability and UV action spectrum are parameters that have benefited from the application of these systems in order to increase the effectiveness and safety of photoprotective formulations containing organic and/or inorganic sunscreens.

## 1. Introduction

Since 4000 BC, evidence exists of the use of cosmetics for beauty care and personal hygiene. The ideal of beauty has undergone several changes over centuries, keeping the desire for the preservation of youth a target. Great attention has always been paid to personal care to maintain youth. The skin is the organ that shows the most visible signs of aging, directly reflecting beauty and youth. The main effects caused by skin aging are wrinkles, pigmentary changes, roughness, flaccidity, telangiectasias, histological and cytological changes induced by ultraviolet rays and hyperpigmentation [1].

Treatment to improve facial appearance, whether with surgeries or with the use of new technologies and dermatological products, has been gradually increasing, not only for reasons of beauty or vanity, but also for social and professional demands. Consequently, new technologies and improved pharmaceutical products appear every day in the search to reduce the aggressiveness of the treatment and, mainly, in order to satisfy the patient. Skin fillers, toxins and rejuvenating pharmaceutical dosage forms are today an important slice of the products available for beautifying and rejuvenating the skin, everyday postponing the exhausting yet effective plastic surgeries [2].

Skin aging is identified over time with notice of signs of changes in its structures [3]. Roughness is one of the most evident signs and can be categorized, according to the causes, into expression lines versus aging lines [4]. Expression wrinkles arise as a consequence of the constant repetition of certain facial movements (such as frowning), while age wrinkles originate due to the loosening of the muscles and the skin itself, influenced by gravity and other factors, such as the actinic damage caused by the sun and cutaneous traumas, leading to a progressive descent of the soft tissues [5].

Other important and easily observed signs in skin aging are weakening and thinning of the skin, dryness, alteration of texture and elasticity with consequent formation of wrinkles, alteration of pigmentation, alteration of vascularization and decrease in the number of attachments (hair, for example). These signs appear throughout the body, but are more apparent on the face, due to greater exposure to the external environment. On the face, the changes that occur throughout life are notable. Anatomically, changes in bone structure and dentition, redistribution of subcutaneous tissue (hypodermis), loss of suspension and skin support mechanisms, changes in facial lines, pigmentation, vascularization and texture, resulting in the appearance of wrinkles and folds [6].

**Figure 1:**
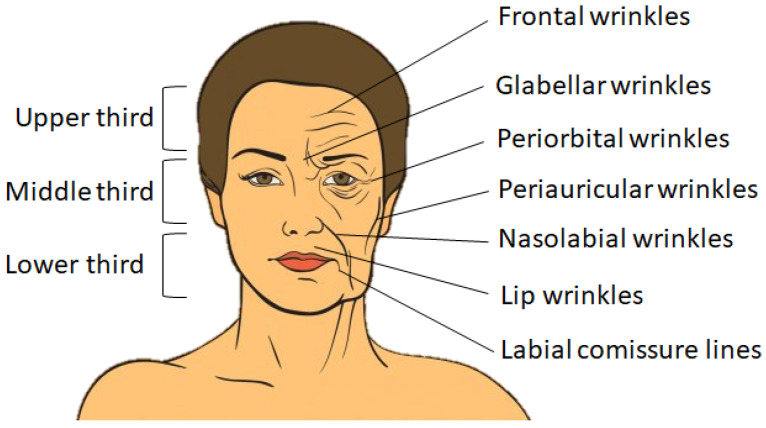
Schematic representation of face segments (left-hand side) and of facial wrinkles affecting the three segments: superior, medium and inferior (right-hand side).

For a better explanation of the changes resulting from aging, the face can be split into three segments, namely the upper third, the middle third and the lower third, as shown in Figure 1 (left-hand side).

In the upper third, changes occur in the configuration of the frontalis, corrugator and procerus muscles. The main signs of aging in this area include forehead wrinkles, falling eyebrows and excess skin on the upper eyelids. In the middle third, there are mainly changes in the lower eyelids, ptosis of the nose and deepening of the nasogenian grooves. Regarding the lower third, the changes are more drastic, with bone resorption of the maxilla and mandible due to loss of dentition and very large sagging of the peri- and sub-labial and sub-labial soft tissues [2], as seen in Figure 1 (right-hand side). 

Generally, wrinkles become more evident in women from the age of 30, when estrogen levels start to decrease and the dermis begins to lose collagen and elastin, becoming more susceptible to the actions of gravity. However, it is from the second half of the fourth decade that the signs of aging intensify, when estrogen levels drop abruptly at menopause and when the renewal of skin cells begins to slow down. Consequently, the epidermis becomes looser and the grooves begin to organize [7]. In addition to these factors, actinic elastosis determined by prolonged exposure to the sun (photoaging), and bone resorption, which occurs mainly after the age of 50, further accelerate this process, which can lead to wrinkles and folds [8].

**Figure 2:**
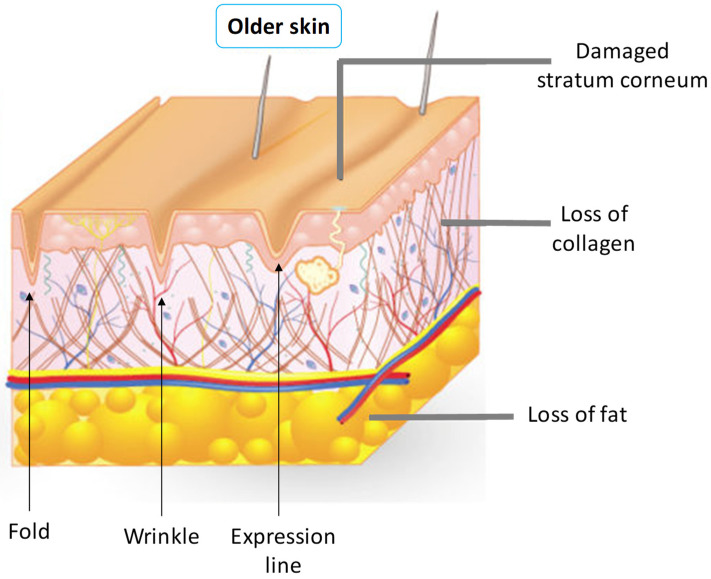
Schematic representation of texture transformations of the skin.

Technically, there are differences between wrinkles and folds. These differences are important when choosing the procedure to be adopted for treatment. Deep wrinkles are the marks left on the skin, superficially in the dermis. The folds are the lines of expression that appear even at rest, marked in the deep dermis. Skin folds occur when the deep folds are covered by a redundant skin from the neighborhood, as shown in Figure 2 [7].

The sun is essential for life on earth and its effects on human health, such as the feeling of physical and mental well-being and stimulating the production of vitamin D and melanin, are just some examples of these benefits to the human body. However, exposure to solar radiation without proper precautions can cause irreversible damage to the body. The effects of exposure to solar radiation depends on several factors, such as (i) individual characteristics of the exposed skin; (ii) frequency and time of exposure; (iii) intensity of radiation, which in turn depends on geographic location, season, time of day and climatic conditions. Thus, the use of adequate photoprotection during periods of prolonged exposure is essential for the prevention of the harmful effects caused by solar radiation. However, providing individuals with photoprotective formulations that allow controlled exposure to solar radiation, in order to obtain only the benefits of radiation, is not an easy task.

The minimum requirements for an efficient and effective photoprotective formulation include: (i) good adhesion to the stratum corneum of the skin; (ii) photostability; (iii) a wide spectrum of action against solar radiation; and lastly, (iv) not causing any type of allergic, cytotoxic or even phototoxic reaction [9]. For the fulfillment of these basic requirements, a photoprotective formulation depends directly on the combination of the sunscreens used in its composition. In this way, new technologies in the area of sunscreens for photoprotection have emerged in recent years, both in the market and in the literature, with the aim of fulfilling these requirements. A very interesting strategy is the use of nanoparticulate systems for topical application of sunscreens. Among several types of nanoparticulate systems, the use of lipophilic biodegradable nanoparticles, which generally contain an encapsulated organic sunscreen, acting as a controlled release device and also as a physical filter by reflection and scattering of light, have bene proposed. Several strategies using nanoparticles as sunscreens in photoprotection have proven to be an excellent alternative in the production of more effective and efficient photoprotective formulations [9].

## 2. The Benefits of Sun Exposure to Human Health

### 2.1. Production of Vitamin D

Sunlight, through ultraviolet (UV) radiation, is an essential part of life and plays an important role in human health. Sunlight has been used as a treatment for diseases, such as vitiligo, *lupus vulgaris*, rickets, psoriasis and tuberculosis, firstly without the real understanding of the mechanisms encountered in the treatment [10,11]. The mechanism of sun exposure is nowadays well established, which also plays an important role in the production of vitamin D [12,13]. Although vitamin D can be obtained from dietary supplementation, UV radiation is the main natural source of vitamin D for humans [13,14,15,16]. 

Most tissues and cells in the human body have receptors for vitamin D and it is estimated that between 200 and 2000 genes may be directly or indirectly under the control of 1,25-dihydroxy vitamin D, or calcitriol, the biologically active form of vitamin D [16]. These genes have as main functions the (i) regulation of cell growth and prevention of malignancies; (ii) increased macrophage phagocytic activity; (iii) modulation of the immune system by changing the ratio of T helper, T helper lymphocytes (Th1 and Th2, important for reducing the risk of autoimmune diseases); (iv) increased insulin production and sensitivity; (v) increased contractility of cardiomyocytes and skeletal muscle function; and (vi) maintenance of blood calcium levels improving the health of populations [16,17,18]. 

**Figure 3:**
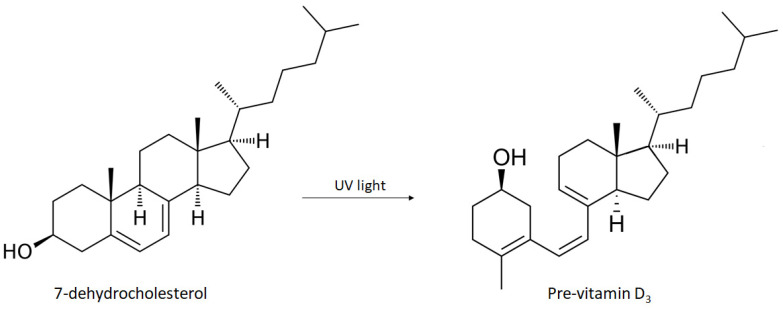
Schematic representation of the synthesis of pro-vitamin D3 from 7-dehydrocholesterol induced by UVB radiation.

The main positive effect of exposure to UV rays on the skin is the conversion of 7-dehydrocholesterol (7-DHC) into pro-vitamin D3 (Figure 3), which is converted by the liver and kidneys into the biologically active form of vitamin D, the calcitriol. 7-DHC is a chromophore that initiates the synthesis of vitamin D in the skin through the absorption of UV radiation, resulting in the formation of pro-vitamin D3. Furthermore, pro-vitamin D3 can be converted to 7-DHC, so excessive sun exposure does not cause intoxication in the body in case of accumulation of pro-vitamin D3. Additionally, pro-vitamin D3 is thermally converted to vitamin D (calciferol), which is biologically inert. To become biologically active, vitamin D requires two hydroxylation steps. The first stage takes place in the liver with conversion to 25-hydroxy-vitamin D3 (25 (OH) vitamin D) under enzymatic control of the 25-hydroxylase, and the second stage takes place in the kidney under the enzymatic control of 1 α-hydroxylase to produce the calcitriol. Circulating levels of calcitriol are maintained by a negative feedback mechanism in the kidneys through the enzyme 1 α-hydroxylase. Therefore, the plasma concentration of 25 (OH) D is a reflection of cumulative exposure to the sun and/or dietary vitamin D intake and is the standard indicator of vitamin D levels in the body [14,19].

The conversion of 7-DHC into pro-vitamin D3 in the skin occurs at a wavelength close to 300 nm, i.e., in the spectrum of UV radiation. Up to 10% of the body’s daily requirement for vitamin D can be obtained through diet (under normal conditions) while approximately 90% is formed from photosynthesis of 7-DHC on the skin’s surface through the action of UV radiation from the sun.

It is estimated that approximately 1 billion people worldwide are deficient in vitamin D, while the largest source of vitamin D production continues to be through sun exposure [15,16]. Regardless of geographic location, circulating levels of 25 (OH) vitamin D are usually quite high in late summer and low in late winter [20]. Controlled sun exposure can provide the proper amount of vitamin D that can be stored in body fat and released during winter, when vitamin D levels are reduced [21,22]. The daily vitamin D levels recommended by the World Health Organization (WHO) are 600 International Units (IU) for children, and 2000 IU for adolescents and adults. Studies have shown that the exposure time for adequate vitamin D production depends on several factors, including (i) time of day, (ii) season, (iii) latitude and also (iv) skin type. In short, it has been shown that exposing arms and legs for a period of 5 to 30 min (between 10 am and 3 pm) twice a week seems to be adequate [21]. This exposure was considered equivalent to the intake of approximately 20,000 IU of vitamin D [23,24].

### 2.2. UV Spectrum

UV radiation is one of the electromagnetic frequency bands [25], originating from the spectrum of total electromagnetic radiation emitted by the sun. The spectrum of solar electromagnetic radiation is composed of UV, infrared (IF) and visible (Vis) light. Visible light covers wavelengths between 400 and 700 nm of the electromagnetic spectrum. UV light is the most energetic component of solar radiation and covers wavelengths between 200 and 400 nm in the electromagnetic spectrum, being the main form responsible for the harmful effects of solar radiation on the skin [26].

Numerous studies demonstrate that UV radiation causes significant cell damage to the skin [26,27,28,29]. An important property of UV radiation that distinguishes it from other radiation is its ionizing capacity and inducing chemical reactions at the molecular level. UV light is a potent environmental mutagen and its interaction with the skin can induce reactions that trigger or exacerbate immune responses to various diseases. However, not all reactions induced by UV light are pathogenic. A good example of this situation is the production of vitamin D from 7-DHC, as previously mentioned. The range of the UV spectrum has been divided into three components based on different biological phenomena, which are related to their respective wavelengths. Thus, the UV spectrum is divided into: UVA between 400 and 320 nm, UVB between 320 and 290 nm and UVC between 290–200 nm [29].

**Figure 4:**
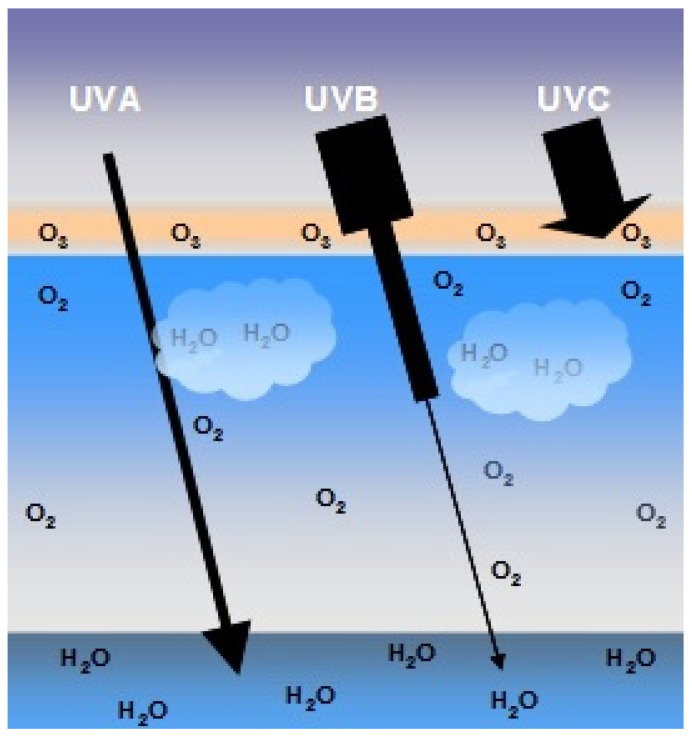
Schematic representation of the penetration of UVA, UVB and UVC rays. Approximately 90% of UVB rays and virtually all UVC rays are absorbed by the ozone layer (O_3_), oxygen (O_2_) from the air and water (H_2_O) in the Earth’s atmosphere. UVA accounts for 95% of the UV radiation that reaches the earth’s surface.

In the Earth’s atmosphere, ozone (O_3_), oxygen (O_2_) and water vapor (H_2_O) selectively filter UVC radiation and most UVB radiation. For this reason, UVA radiation corresponds to approximately 95% of the UV radiation that reaches the Earth’s surface (Figure 4). Approximately 90% of UVB radiation is absorbed by ozone contained mainly in the terrestrial stratosphere. However, since the constant emissions of chlorofluorocarbons (CFCs) alter the density of the ozone layer, a larger portion of UVB and UVC radiation may reach the Earth’s surface. Although UVB radiation reaches the Earth’s surface in a small proportion, its energy is much higher than UVA radiation. It is estimated that UVB radiation is 1000 times more erythematogenic than UVA radiation, i.e., even though UVA radiation reaches the Earth’s surface in greater concentration, UVB radiation is the main form responsible for erythema and/or redness and mainly for burns in the skin. However, unlike radiation energy, the ability to penetrate the skin is proportional to the radiation’s wavelength. Thus, UVA rays that have a longer wavelength also have greater penetrating power. UVA radiation can penetrate the deepest layers of the dermis, while UVB rays are only absorbed up to the epidermis [26,30]. 

Exposure to solar radiation is usually assessed in terms of its form of exposure, i.e., it can be recreational or occupational, in an open or closed environment. Factors related to social and cultural behavior, such as clothing preferences and exposure habits, are also responsible for significant variations in exposure to solar radiation between different groups. In addition, environmental factors, such as time (UV radiation is most intense between 10 and 16 h), latitude (the closer to the equator the higher the radiation levels), altitude (the intensity of UV radiation increases from 10 to 12% every 1000 m), weather conditions (sky covered by a thick layer of clouds can reduce the levels of UV radiation), interaction of surfaces with UV radiation (soil and water reflect less than 10% of UV radiation, the snow reflects up to 80%, beach sand reflects 15% and sea foam reflects 25%) and the thickness of the ozone layer (variations in thickness must be observed through variations in the UV radiation index) are relevant factors to consider in the history of individuals regarding exposure to solar radiation.

As previously seen, UVB radiation is more energetic than UVA radiation, being the main form responsible for sunburn, tanning and photocarcinogenesis caused by excessive sun exposure. UVB radiation is the main cause of (i) damage to deoxyribonucleic acid (DNA), (ii) induction of inflammation, (iii) immunosuppression as well as (iv) the synthesis and release of prostaglandins (PG—particularly PGE2) through of the induction of the cyclooxygenase-2 (COX-2) enzyme. UVB radiation also induces ornithine decarboxylase, an enzyme that limits the speed of the biosynthesis of polyamines that stimulate cell proliferation, contributing to the formation of carcinomas. They also stimulate the enzyme nitric oxide synthase that induces erythema [31], melanogenesis and contributes to immunosuppression [32]. UVB radiation also affects cutaneous angiogenesis by decreasing the expression of the thrombospondin-1 angiogenesis inhibitor by inducing the expression of vascular endothelial growth factor (VEGF, Vascular Endothelial Growth Factor) and endothelial cell growth factor 1 (ECGF1, Platelet- derived endothelial cell growth factor) [33,34,35].

Last but not least, UVB radiation promotes the migration of leukocyte-producing elastase in the skin, aggravating the degradation of elastin. Despite the exhaustively reported harmful effects of UVB radiation, UVA radiation is suspected to be responsible for proportionally developing a broad role in photoaging. The fact that UVA radiation is present on the Earth’s surface in a proportion 10 times higher than UVB radiation results in a higher average irradiation and with a deeper penetration in the epidermis and dermis when compared to UVB radiation, which makes UVA radiation one of the main forms of radiation responsible for the mechanisms that involve premature aging. There are recent studies that show that skin exposed daily for a period of one month to doses of sub-erythogenic UVA radiation demonstrates signs of epidermal hyperplasia, thickening of the stratum corneum, depletion of Langerhans cells and inflammatory infiltrations in the dermis with deposition of lysozymes in the elastic fibers [36]. UVA radiation also induces the synthesis and release of cytokines and matrix metalloproteinases (MMPs), particularly collagenase (MMP-1) and elastase, as well as heme-oxygenase-1, an enzyme responsible for raising the levels of intracellular iron promoting the generation of additional reactive oxygen species (ROS) [28,32,37]. In addition, UVA radiation also triggers mitochondrial DNA mutations [36,38,39]. Both UVA and UVB lead to the generation of ROS, which cause damage to cell lipids, proteins and DNA [38,40]. 

### 2.3. Photoaging

The skin aging process includes progressive physiological changes in the body that lead to senescence, which is defined as the decline in biological functions and the body’s ability to adapt to metabolic stress over time. The aging process is complex and multifactorial, resulting in several functional and aesthetic changes in the skin. These changes are the result of both intrinsic (chronological) and extrinsic factors, which are related to environmental factors, mainly the damage caused to connective tissues in the skin dermis by UV radiation. There is evidence that the intrinsic and extrinsic aging processes partially overlap the biological, biochemical and molecular mechanisms of aging [37,40,41].

By definition, photoaging is the overlapping of the chronic effects of UV radiation on intrinsic aging that results in age-associated changes in the appearance of the skin. These changes are triggered by signals mediated by receptors, mitochondrial damage, oxidation of proteins and telomeres and in the control of response pathways to DNA damage [40,41,42].

Today, more people are looking for treatments to reverse the processes associated with skin aging. The perception of aging associated with beauty is largely dependent on the appearance of the exposed skin, and its condition is dependent on the effects of the environment, especially UV rays.

The skin damage produced by sun exposure varies considerably between individuals, and directly reflects the inherent differences in vulnerability and in the skin’s ability to repair damage caused by sunlight. Even among Caucasians, the appearance of the photodamaged skin of individuals with skin types I and II, often differs from that of individuals with skin types III and IV (Table 1). In general, photodamaged type I and II skin show atrophic changes, such as loss of skin thickness (appearance of thin and wrinkled skin) with few wrinkles and sometimes focal depigmentation (idiopathic gut hypomelanosis) and dysplastic changes, such as actinic keratosis and epidermal malignities. In contrast, hypertrophic changes, such as deep wrinkles, thick skin, armored-looking skin and lentigo (brownish skin color changes, which usually occur on the back of the hands, neck and face) appear in individuals with type III skin and IV [37,42]. 

A characteristic of photoaged skin is elastosis, clinically identified as a discoloration of the skin to brownish yellow with a rough surface. Histologically, the dermis is shown as a tangle of degraded elastic fibers, as well as an amorphous mass composed of disorganized tropoelastin and fibrillin. In addition, the amount of connective tissue, mostly composed of glycosaminoglycans and proteoglycans (polysaccharides that assist in the maintenance of skin tissue), is elevated and deposited incorrectly in the photodamaged skin, while the amount of collagen is reduced. In addition, there is a greater number of hyperplastic fibroblasts as well as an increase in inflammatory cells, including mast cells, histiocytes and other mononuclear cells, resulting in heliodermatitis (skin inflammation due to sun exposure). The dermal vasculature on gently photodamaged skin shows thickening of the vein wall; in severely photo-damaged skin, the walls of thin vessels have compromised perivascular cells and present dilations (telangiectasias) [41,42,43,44,45].

## 3. Skin Cancer

In recent years, there has been an increase in exposure to solar radiation by the general population. This increase in exposure to solar radiation is the main reason for the increased number of cases of skin cancer [46]. 

The link between exposure to solar UV rays and skin cancer is an old one and was described by Unna and Dubreuilh at the end of the last decade of the 19th century [47]. The authors observed actinic keratoses and squamous cell carcinomas on the skin chronically exposed to solar radiation from sailors and vineyard workers. Currently, numerous epidemiological and scientific studies are showing that exposure to sunlight causes cancer [14,30,48].

Skin cancers are mainly categorized as melanoma and non-melanoma skin cancers, including the latter the basal cell and squamous cell cancer. Melanoma is responsible for the largest number of deaths from skin cancer, while non-melanoma cancers are typically described as having a more benign course and aggressive local lesions [28]. 

According to WHO data, an estimated 60,000 deaths per year are a consequence of exposure to UV radiation. Of these 60,000 deaths, an estimated 48,000 are due to malignant melanoma and 12,000 are skin carcinomas [49].

As we saw in the previous sections, UVB radiation constitutes only 4–5% of UV radiation, but it is nevertheless the most harmful, causing damage that includes (i) sunburn, (ii) inflammation, (iii) DNA damage, (iv) oxidative stress, (v) free radical production, (vi) immunosuppression, (vii) photoaging and finally, (viii) skin cancer.

UVB solar radiation induces the development of skin cancer through a process that involves three distinct stages that can be exemplified as initiation, promotion and progression. Each of these stages are mediated by several changes at the cellular, biochemical and molecular level. The initiation stage is the first step in the process of photocarcinogenesis and involves genetic changes that lead to mutations in the DNA of normal cells, and is essentially an irreversible process. The tumor promotion stage involves clonal expansion of the mutated cells by changes in transduction pathways and is considered reversible. The tumor progression stage involves the malignant transformation of papillomas into carcinomas [34,39,50]. Since the initiation process occurs quickly, strategies to avoid the intervention initiation process are difficult to envisage. Skin cancer induced by solar radiation can take years or decades to develop and the best time for intervention appears to be in the stages of promoting or progressing carcinogenesis, since both stages are slow [50].

In general, approximately 20 to 30% of skin cancers are diagnosed in Caucasians, 2 to 4% in Asians and 1 to 2% in blacks and Asians of indigenous origin. In 2006, of all skin cancers, melanoma accounted for approximately 1 to 8% of cases in blacks, 10 to 15% in Asians of indigenous origin and 19% in Japanese. Even though skin cancers are not prevalent in black-skinned individuals, they may have greater morbidity and fatality as they are not easily and timely diagnosed [51].

As the incidence of skin cancer has been increasing at alarming rates, it has become one of the biggest challenges for public health systems. The pathogenesis of skin cancer is multifactorial; however, UV radiation is the biggest factor that contributes to the development of the disease.

## 4. Sun Protection Factor

The effectiveness of a solar protector is evaluated according to its SPF to UVB radiation, which indicates how many times the time of exposure to the sun, without the risk of erythema, can be increased with the use of the protector [52]. Considering the same geographic locations, seasons, weather conditions and time of day, a light-skinned person (type II skin) can be exposed to the sun for 15 min without sunscreen, he can be exposed to the sun for 225 min with an SPF protector. = 15, since 15 × 15 = 225. The higher the SPF, the greater the protection, that is, the longer the skin will be protected from UVB radiation. The FPS value is calculated using Equation (1) [53]:

(1) $$\mathrm{SPF}=\frac{\mathrm{Minimal}\;\mathrm{erythematous}\;\mathrm{dose}\;\mathrm{on}\;\mathrm{skin}\;\mathrm{with}\;\mathrm{protection}}{\mathrm{Minimal}\;\mathrm{erythematous}\;\mathrm{dose}\;\mathrm{on}\;\mathrm{skin}\;\mathrm{without}\;\mathrm{protection}}$$

For the evaluation of the SPF, special attention must be paid to the need for the correct application of the product on the skin. According to the quantitative standard of sun protection per skin unit, it is necessary to measure the SPF at 2 mg/cm^2^ in humans [54]. Thus, each application must comprise an amount of 30 to 40 g of the product by an adult individual, of normal size and weight. Studies have shown that the application of protectors containing inorganic filters are more compact and as such, they become more difficult to spread when compared to protectors containing organic filters. These considerations are reinforced by some studies carried out with consumers, which indicate that the SPF obtained without following the quantitative procedure mentioned above results in values that can reach one third of the proposed value [55].

In August 2007, the Food and Drug Administration (FDA) published the final monograph with the new guidelines in relation to sunscreens used in the formulations of photoprotectors, as well as the mandatory performance of photostability, UVB and UVA photoprotection tests in vivo and the creation of a UVA protection classification. In this sense, and with the aim of offering preparations with greater efficiency (products with better protection efficiency, greater chemical stability and more accessible to the population), the segment has demanded from the industry better technical improvement and quality in the acquisition of raw materials for the development of new sunscreens. In addition, a better understanding of the physical–chemical behavior of new and traditional molecules used as sunscreens is needed. In this review, we discuss some options that nanotechnology can offer in the area of photoprotection through the manufacture of sunscreens at a nanometric scale. The importance of nanometric properties, phenomena that involve their interaction with light, protection mechanisms and interaction with biological systems are addressed.

## 5. Lipid Delivery Systems for Dermocosmetics

Expression wrinkles and deep wrinkles are usually treated in a non-surgical manner with the application of botulinum toxins, creams that penetrate the epidermis and smooth these wrinkles and with the use of skin fillers, such as hyaluronic acid and polymethylmethacrylate (PMMA). The folds are deeper grooves and eventually need to be treated with surgery. However, with the advancement of possibilities, most of the time they are treated with thicker skin fillers associated or not with botulinum toxin. Skin folds have no other therapeutic option than surgery, since, due to the observed redundancy, even thick fillings are not able to correct the deformity, so surgical resection of the excess skin is necessary [56].

In recent years, the encapsulation of bioactive molecules in colloidal lipid-type systems has received great attention for topical treatment [57]. The main benefits obtained with the use of delivery systems for topical use include the improved capacity to transport bioactive molecules and the ability to overcome skin barriers [58], promoting the bioactive stability and minimizing skin irritations [59], promoting the controlled and/or sustained release of the bioactive molecule [60,61,62], improving spreadability of the formulation and, consequently, greater patient compliance. In addition, lipid nanoparticles provide hydration [63], enhanced viscosity [64,65] and skin occlusion [66]. These benefits are governed by the type of lipid nanoparticles, their composition and the bioactive molecules to be loaded [67]. In addition, it can be noted that this non-invasive route of administration offers numerous advantages, among which the escape to the first-pass phenomenon of hepatic metabolism, direct application to the target organ (skin) and due, to easy application and absence pain, a greater patients’ compliance to treatment [68].

**Figure 5:**
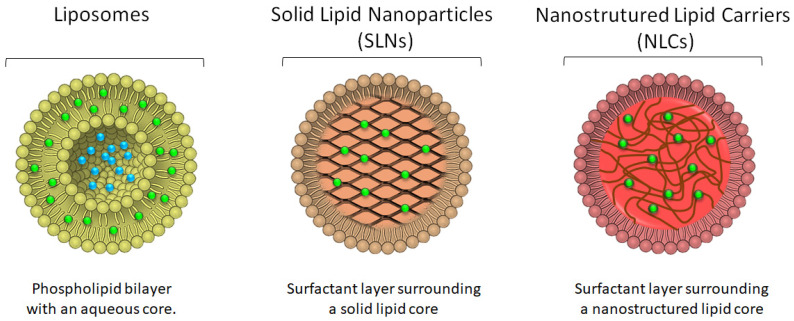
Schematic representation of the morphology of liposomes, solid lipid nanoparticles and nanostructured lipid carriers, depicting the location of the drug (blue dots for hydrophilic drugs and green dots for more lipophilic drugs) in each of the systems.

It should be noted that lipids are excipients that are part of the physiological structure of the skin, among the corneocytes we find ceramides, cholesterol and fatty acids. In the deeper layers of the epidermis, we find more polar lipids, such as triglycerides and phospholipids [69]. The use of free fatty acids provides acidification of the skin surface, preventing the action of pathogens. The presence of cholesterol also regulates the rigidity of lipid membranes, and ceramides are the most important class of lipids in the intra-corneocyte space [70,71]. Among the lipid nanoparticles described in the literature, this section will focus on liposomes [72,73] and lipid nanoparticles (SLN and NLC) (Figure 5) [63,68,74].

### 5.1. Liposomes

Liposomes resemble cell membranes, i.e., they are formed by spherical vesicles containing one or more layers of phospholipids. This type of lipid nanoparticles acts as a reservoir of bioactive molecules with potential to achieve sustained release. In addition, the reservoir effect decreases the risk of the drug to reach systemic circulation via transdermal route, and minimizes the undesirable effects [75].

Examples of bioactive molecules already loaded in liposomes include benzophenone [72], glycolic acid [76], o magnesium ascorbyl phosphate, alpha lipoic acid and kinetin [73], the octyl methoxycinnamate [77], curcumin [8,78], 5-aminolevulinic acid [79], cocoa, green tea and alpha-tocopherol [80], co-enzyme Q10 [81,82], ursolic acid [83] and vitamin A [84]. As depicted in Figure 5, hydrophilic drugs will be placed in the inner aqueous core of the phospholipid bilayer, whereas lipophilic/hydrophobic drugs will be preferentially located within the hydrocarbon chains of the bilayer.

Lee and Tsai (2010) [82] studied the encapsulation of coenzyme Q10, a potent antioxidant, in liposomes in order to reduce the effects of skin aging. The liposomes were produced with soy phosphatidylcholine and vitamin E. The results showed that the lipid vesicles had a size around 200 nm and a polydispersity of about 0.3. In vivo studies were performed in rats and for this purpose, two formulations were used, coenzyme Q10 in solution and coenzyme Q10 encapsulated in liposomes. The results showed that the liposomal form obtained significantly greater cutaneous penetration than the solution. Thus, the liposomal formulation has been shown to be able to increase the concentration of coenzyme Q10 in the skin and maintain it for a longer period of time.

Manconi et al. (2011) [85] developed liposomes for encapsulation of retinoic acid. In addition to soy phosphatidylcholine, hydrophilic excipients (Oramix^®^ NS10, Labrasol^®^, Transcutol^®^ P and propylene glycol) were added to increase skin penetration. The results showed that the vesicles had a size around 150 nm, polydispersity 0.3 and negative zeta potential (−55 mV), with an encapsulation efficiency of approximately 87%. The influence of hydrophilic excipients was evaluated in ex vivo studies in pig skin, and the results showed that there was a greater accumulation of retinoic acid in the epidermis and less in the dermis. In addition, scanning electron microscopy studies have shown evidence of the ability of liposomes to interact with intercellular lipids.

Curcumin is a natural polyphenol obtained from the roots of Curcuma longa and has antioxidant action and prevents lipid peroxidation. This bioactive has pharmacological action, such as anti-toxicity, anti-cancer, anti-inflammatory, anti-wrinkle and anti-viral effects [86]. Currently, curcumin is marketed in the form of creams and gels (Vicco Turmeric^®^ cream and Emami Gold^®^), but has low bioavailability on the skin [87]. Gupta and Dixit (2011) [8] encapsulated curcumin in liposomes and niosomes, followed by incorporation in carbopol gel. The in vivo results showed that the formulations containing the encapsulated curcumin increased the antioxidant effect and the anti-aging effect. The researchers concluded that this increase in anti-toxicity was due to the amphiphilic nature of the lipid vector, promoting an increase in the miscibility of curcumin. Thus, the efficiency of phospholipid vesicles in the anti-aging, antioxidant and anti-wrinkle action was clear.

### 5.2. Lipid Nanoparticles

In the last 30 years, numerous nanoparticulate systems of organic origin have been proposed in the literature as carriers of sunscreens for application in photoprotection. Systems containing organic nanoparticles are considered safer for use in humans and more environmentally friendly, emerging as an alternative to systems that generally use titanium dioxide and zinc oxide [88,89]. The main examples of these systems are the solid lipid nanoparticles (SLN, Solid lipid nanoparticles) and nanostructured lipid carriers (NLC, Nanostructured lipid carriers). Given their lipid composition, SLN and NLC are preferentially suited for the loading of lipophilic compounds within their matrices (Figure 5).

#### 5.2.1. Solid Lipid Nanoparticles (SLN)

SLN are composed solely of a solid lipid, whereas NLCs are lipid nanoparticles composed of a mixture of solid lipids and liquids; both SLN and NLC stabilized by surfactant molecules. They present occlusive properties due to the formation of a lipid film that forms on the skin, reducing water evaporation and promoting hydration [63,90,91,92].

The idea of using SLN as a sunscreen was introduced shortly after observing in a previous work with SLN a pigmenting and bleaching behavior similar to that found in titanium dioxide nanoparticles [92,93]. Further research was undertaken to test in vitro the absorption capacity of SLN formulations compared to nanoemulsions with and without DL-α-tocopherol acetate, using the Transpore^TM^ tape methodology. The UV light absorption results of the placebo SLN were 100% higher than the placebo nanoemulsion formulation at the same concentration. The authors concluded that the significant absorption shown by SLN was due to the reflection and scattering of UV light, since the components used in the formulations do not absorb UV light at the analyzed wavelengths. SLN containing DL-α-tocopherol acetate showed better performance in the absorption of UV light, mainly at wavelengths below 300 nm, the absorption region of DL-α-tocopherol acetate. The increase detected in UV absorption below 300 nm in the SLN formulation containing DL-α-tocopherol in relation to the SLN placebo represents, according to the authors, the synergistic effect of the combination of chemical and physical molecular sunscreens in blocking UV light. 

In another work, Wissing et al. used the sunscreen benzophenone-3 (BZ-3) as a model of organic chemical filter incorporated in the SLN [66]. In this case, the synergistic effect caused by the association of SLNs containing 1% BZ-3 can be observed in vitro by comparing the absorption curve of the combination (SLN containing 1% BZ-3) with the absorption curve of the placebo SLN, that the theoretical absorption curve of BZ-3 in 1% solution was added. The absorption found in the association of SLN with the organic filter BZ-3 at 1% was greater than the sum of the absorption of the SLN placebo with the theoretical absorption of BZ-3 in 1% solution.

This same synergistic effect can be observed when comparing the absorption capacity of SLN and emulsions consisting of 20% lipid matrix with different concentrations of BZ-3 [67]. SLN absorbed UV radiation, on average twice as much as emulsions.

In addition, the use of SLN in association with the organic sunscreen allows to reduce the amount of sunscreen in products already commercialized by 50%, maintaining the same level of absorption [94,95,96]. This fact clearly demonstrates the importance of SLN in the synergistic effect with organic filters in blocking UV light. Synergism allows the reduction of the concentration of organic filters used in photoprotective formulations, thus reducing the toxicological risks of prolonged exposure while maintaining the same levels of photoprotection.

The reflection and spreading properties of SLN are directly related to the crystallinity of the lipid matrix [94]. The greater the organization at the molecular level of the lipids involved, the greater the packaging of the lipid matrix and the more crystalline the matrix will be [97]. However, the particle size and lipid concentration present in SLNs also directly influence the blocking properties of UV light [98]. SLN with diameters between 100 and 200 nm were more effective in blocking UV light compared to particles of the same composition of micrometric sizes (4.6 μm). These results were confirmed by testing the UV light blocking capacity of polystyrene particles with different sizes. The SLN with particle sizes between 500 and 1000 nm were the ones that showed the best results in blocking UV light. In addition to size, the lipid concentration (10–40% cetyl palmitate) also influenced the blockade, i.e., the higher the lipid concentration in the SLN, the greater the blockage.

Another important parameter in a photoprotective formulation used to block UV light is the ability to form a uniform film after application to the skin surface. In vitro tests demonstrate that the use of SLN in photoprotective formulations allows the formation of a more homogeneous and uniform film on the skin surface, in addition to allowing the control of the properties of the formed film through the manipulation of the particle size, concentration and crystallinity of the lipid phase [67,97,99,100]. The achievement of a more uniform film using nanoparticles is mainly due to the small particle size and the resulting large surface area. The nanometric particles, when spread on the skin, cover a superior surface area and more uniformly than micrometric particles. In this way, the application of a micrometric formulation forms a film with larger pores, allowing the passage of UV radiation more easily compared to nanometric formulations, where the pores are smaller in size. Consequently, the protection against the passage of UV radiation is more efficient using nanometric particles. 

The homogeneity of films containing SLN was analyzed through the absorption of UV light using Transpore^TM^ tape [98]. A dispersion of SLN consisting of 10% cetyl palmitate, 1.2% polyglyceryl methylglucose distearate (Tego Care 450) and water was applied uniformly to the surface of the Transpore^TM^ tape with the corresponding area being 4.5 cm^2^. After drying, the tape was fixed to a quartz cuvette and the light absorption from 250 to 450 nm of the film formed in different positions was analyzed. The results revealed a slight variation in the absorptions in the UV spectrum along the tape, which indicates a uniformity of the film formed on the surface of the Transpore^TM^ tape by the SLN, which consequently also suggests a uniformity in the blocking of UV light.

More recently, with the aim of developing safer photoprotective formulations, a combination of chitin (a natural component with good biocompatibility) and the chemical UV filter (3,4,5-trimethoxybenzoylchloride), formed from the chemical reaction compound 3,4,5-trimethoxybenzoyl chitin (TMBC) was developed as a new molecule for absorption of UV radiation [101]. TMBC was incorporated in SLN with the aim of reducing the harmful effects of the chemical filter and increasing the protection spectrum in the UVB. The effectiveness of the absorption of UV radiation from the sunscreen when incorporated in SLN (prepared with glyceryl monostearate) was significantly higher. Finally, 5% tocopherol was added to the sunscreen and the absorption spectrum between 200 and 450 nm was compared with formulations containing only SLN and 5% TMBC, or only, SLN and tocopherol. In this study, there was a visible increase in absorption in the UVB spectrum, between 250–325 nm, for the SLN formulations with TMBC and tocopherol. As a result, the authors infer that the toxicity of the original chemical filter has been reduced and that this new system will be more acceptable for topical application, with greater liposolubility, which makes it possible for SLN to transmit TMBC and act simultaneously as a physical filter. The authors also infer that TMBC could potentially be used as a sunscreen, since it does not dissolve in water, in addition to acting in synergy with SLN, significantly increasing the UV absorption spectrum [101].

In another study, the organic sunscreen bis-ethylhexyloxyphenol methoxyphenyl triazine (BEMT) with a broad absorption spectrum (280–390 nm) was incorporated into the SLN [102]. Despite its broad spectrum of UV absorption, its reduced solubility in several solvents and reduced photostability limit the use of BEMT in photoprotective formulations. However, BEMT encapsulation efficacy in SLN proved to be satisfactory compared to the lipid matrix of placebo SLN and SLN containing 5% TMBC, allowing a significant increase in the photostability of the organic filter in the formulations produced. As for the study of chemical photostability of BEMT, SLN proved to be effective in protecting the molecule when exposed to radiation for 12 h. In this case, the authors demonstrate that SLN can also help to increase the photostability of organic filters when encapsulated in the lipid matrix.

#### 5.2.2. Nanostructured Lipid Carriers (NLC)

In addition to the encapsulation of organic filters, it has also been proposed to use inorganic filters encapsulated in the SLN lipid matrix [103,104] and NLC [105,106,107]. Some authors have proposed the encapsulation of inorganic sunscreens, such as barium sulfate, strontium carbonate and titanium dioxide in lipid matrices composed of carnauba wax and decyl oleate [105]. While the average SPF of nanosuspensions produced with only 6% titanium dioxide dispersed in the aqueous phase was around 4, when the filter was incorporated in the lipid phase it was possible to reach SPF close to 50. The increase in SPF caused by the association of carnauba wax with titanium dioxide is, according to the authors, due to a synergistic absorption effect between the delocalized electrons of the π orbitals contained in the cinnamate derivatives of the carnauba wax with the continuous absorption band formed by the nano-agglomerates of dioxide titanium in the lipid matrix [106]. Considering the semiconductor character of titanium dioxide, the more units of titanium dioxide that approach in a cluster, the more continuous the conduction band formed and the less energy required to promote the electrons of the valence band to the conduction band. In addition, the transfer of electrons from the π orbitals displaced from cinnamates to the conduction band of titanium dioxide under irradiation allows for an even greater increase in UV absorption and, consequently, SPF.

Following the trend of the new generation of lipid carriers, namely NLC, numerous studies with organic sunscreens have been carried out demonstrating their considerable capacity for encapsulation and absorption in UV [108], its physicochemical stability [109] and, more specifically, its synergistic effect [110].

A comprehensive study carried out with NLC prepared from mixtures of lipids of different natures with organic sunscreens, reported that not only does the physical state of the lipid (solid or not) affect the synergistic effect, but also the chemical nature of the lipid matrix of the NLC [110]. The concentration of organic sunscreen was maintained, while different lipid matrix compositions were tested. The NLC formulations produced were analyzed for their physical–chemical stability, light absorption in UV and SPF. It was found that in some cases, the UV absorption of NLC composed of certain lipid matrices showed similar and/or even lower values than the reference emulsions. According to the authors, this difference in the absorption behavior of NLC may be related to the location that the sunscreen adopts in the matrix. There are three models of localization of pharmacologically active substances (PAS) possible in the NLC matrices, in the first the PAS is internalized in the nucleus (core) of the particle, in the second it is located on the surface (shell) or lastly, it is if molecularly dispersed in the matrix [97]. In cases where the NLC absorption was lower or similar to the reference emulsion, it is deduced that the most likely model would be the first, i.e., the filter would be located in the particle’s nucleus, since they would not be absorbing UV light and absorption values found would be derived solely from the reflection and scattering of the NLC particles. For the models where the filters would be on the particle surface, the synergistic effect between the UV absorption of the sunscreens with the reflection and spreading of the NLC is observed, both for the UV light absorption studies and for the SPF in vitro studies. 

Considering that sunscreens are applied more frequently and for longer periods, their safety and effectiveness are fundamental factors and must be considered when formulating a photoprotector. The ideal sunscreen should be effective at low concentrations, photostable and remain on the surface of the epidermis, avoiding penetration into the deeper layers of the skin, such as the dermis, which can cause unwanted systemic absorption [111]. The presence of sunscreen on the surface of the stratum corneum allows greater effectiveness in blocking UV light preventing phototoxic and photoallergic reactions often observed after application of organic sunscreens [112,113].

The authors used two different cell models, mouse embryonic cells and human keratinocyte cells [114]. In vivo allergenicity was assessed using mice as a model. No allergic, cytotoxic or even phototoxic effects were detected in the tests performed using the SLN placebo and BZ-3 formulations, demonstrating their biocompatibility and safety. Regarding both in vitro and in vivo studies of the evaluation of SPF, they demonstrated that the formulation of SLN with BZ-3 was, on average, three times more effective in protection.

Since the filters should only be on the surface of the epidermis, the studies evaluated the in vitro and in vivo permeation of sunscreens in SLN formulations with BZ-3 [66]. Two studies were carried out to evaluate permeation in vitro, namely the free membrane model and through Franz diffusion cells. The release profiles of BZ-3 from SLN were compared with those of emulsions and it was found that the release profile of emulsions was faster than SLN. This effect may be due to the fact that SLNs present a solid matrix, i.e., more crystalline, modifying the release profile. In the case of systems intended for photoprotection, this effect is desired, since more filter remains on the skin surface. Another observed result was the fact that higher concentrations of BZ-3 result in a slower release of the filter from the SLN. In this way, the greater the concentration of the filter on the skin surface, resulting in a greater protection factor [115]. The results of both methods used correlated that the release of BZ-3 is sustained and is lower in SLN and when higher concentrations of filter are used. This can be explained by the location of the BZ-3 molecules in the lipid matrix and also by the Christianity of the SLNs in relation to emulsions, resulting in a delay in the diffusion of the filter from the interior of the particles to the membrane surface. It was also observed that SLN forms a film on the skin after its application, resulting from its fusion with the lipids present in the skin, after the evaporation of the water in the formulation. Still in the same work, in vivo permeation studies were also carried out using the adhesive tape technique in order to correlate these results with the results obtained in vitro [116]. The permeation results of BZ-3 in the skin for the emulsions incorporated in the concentrations of 5 and 10% were, respectively, 50% and 10% higher than the SLN in the same concentrations. The permeation of BZ-3 is an undesired effect in view of the potential phototoxic, allergenic interactions and systemic effects. As demonstrated, permeation is dependent on the concentration of BZ-3 employed in the formulation. Through these results, the authors suggest the potential use of SLN as a physical filter so that the concentration of potentially toxic molecules can be reduced in cosmetic photoprotective formulations, while maintaining the same sun protection factor. SLNs are capable of providing sustained release, allowing the sunscreen to remain on the skin surface longer. Furthermore, the use of higher concentrations of BZ-3 results in decreased release and permeation.

The occlusion factor for lipid microparticles with a diameter of 1 µm is only 10%. However, for NLC, which had an average size of 200 nm, they have a factor of 50%. The NLCs produced with the pure lipids tripalmitin and tristearin, promoted occlusion and high hydration. Using lipids with a low melting temperature, such as tricaprine and trilaurine, low occlusion is obtained. The hydration of the skin can be easily measured in vivo by means of a corneometer, measuring the electrical capacity of the skin before and after the applications of the formulations [117].

Besides the occlusion effect of lipid carriers that increases skin hydration and permeability, the degree of drug penetration through the skin is also governed by the type of lipid carrier, its lipid composition and mean size. Sunscreens of organic and inorganic origin should remain onto the surface of the skin without increased bioavailability as they should not reach deeper skin layers. On the other hand, antioxidants are expected to permeate deeper into the skin. The overcome of skin barrier integrity has been attributed to an intrinsic mechanism related to specific interaction between drug and lipid carrier, together with the skin surface [60].

Souto and Muller (2008) described a study with volunteers to compare the hydration of the skin when applying a cream day and night, and after having replaced a part of the oily phase of the cream with NLC [63]. The high-quality cream used in this study showed high hydration effects, that is, the addition of NLC actually promotes skin hydration. Although studies of this type do not fully mimic the natural conditions of moisture loss, the smaller the particle size, the better the barrier to avoid evaporation, since the surface area of the particles is larger, making it impossible for water to escape.

The typical occlusion produced by the formulations does not ensure rapid hydration, particularly if the skin is excessively dry, so it is desirable to use a preparation capable of providing water. NLC dispersions are suitable for this purpose when applied to the skin, since the pressure exerted leads to the fusion of the particles that form a dense film [66,98]. This fusion is promoted by the capillary forces involved during the evaporation of water [67]. The formation of this film was confirmed by scanning electron microscopy [63].

NLCs also promote adhesive properties when they are in contact with surfaces [118]. The spherical shape of the particles provides a degree of adhesion depending on their average diameter and can be easily evaluated by analyzing the texture. With similar sizes, the NLCs can be compared with respect to their viscosity surface properties, and can be incorporated into pharmaceutical forms, such as, for example, gels, creams and lotions. In the case of hydrogels, what gives viscosity is the carbopol or polyacrylate hydrogels that have incorporated NLC [119].

In the prevention of wrinkles, the daily use of sunscreen stands out. NLC are used both for the encapsulation of sunscreens of organic and inorganic origin, and are also efficient to increase the sun protection factor (SPF) of cosmetic formulations [104]. Examples include benzophenone [120], zinc oxide and octocrilene [121] and ethylexyl methoxycinnamate [122].

Lacatusu et al. (2011) [123] studied the effect of octocrylene sunscreen encapsulation in NLC. The study evaluated the photoprotection index and the stability of the formulation. The in vitro determination of the SPF obtained was 20, being able to filter 95% of the ultraviolet rays. The photoprotection of NLC formulations increased by two compared to conventional formulations. Similar results, showing an increase in solar SPF, were also observed by the authors [66,103,108].

The bioactive molecules carried on NLC for the treatment of wrinkles already reported in the literature include retinoic acid [59,124,125], octyl methoxycinnamate [126], the co-enzyme Q10 [127], lutein [128], beta-carotene and alpha-tocopherol [92,129], ascorbyl palmitate [130], the idebenone [131], resveratrol [132] and curcumin [133].

Plianbangchang et al. (2007) [133] studied the encapsulation of curcumin in NLC. This study aimed to encapsulate curcumin in NLC using the microemulsion method and later incorporating it into a cream for facial application. The formulation was studied in 33 volunteers. The evaluated results were carried out before and after the treatment, which lasted 8 weeks. The parameters evaluated include the appearance of wrinkles, hydration, the amount of melanin, elasticity, viscoelasticity and irritation. The results revealed that NLCs incorporating curcumin, from the third week of treatment on, were significantly more effective in reducing wrinkles and in improving hydration, elasticity, melanin content and viscoelasticity, compared to the control cream. In addition, there were no cases of skin irritability.

Coenzyme Q10 is an antioxidant used in the treatment of wrinkles. Due to the instability of this molecule, its encapsulation becomes a viable alternative to promote its bioavailability. The encapsulation of coenzyme Q10 in NLC by the high-pressure homogenization method was studied by Farboud et al. (2011) [127]. The results showed that the NLC obtained a size that varied from 50 to 100 nm, with prolonged release compared with the free coenzyme Q10. The formulation was evaluated in 25 volunteers suggesting that coenzyme Q10 showed good penetration in the dermis with coenzyme Q10 activity in the skin promoting hydration and the anti-wrinkle effect superior to conventional formulation.

## 6. Commercialized Formulations

In general, the rules for applying bioactive molecules and colloidal vectors for topical use are less strict when compared to those aimed for systemic administration, making it faster to launch an innovative product on the market for cosmetic use. In 1986, the first liposomal cosmetic product for the treatment of aging appeared on the market, called Capture^®^, launched by Dior. Soon after, in 1987, L’Oreal launched a product called Niossome^®^. Since then, a range of new products have been launched for the treatment and prevention of skin aging, including wrinkles. More examples of products marketed with this technology include the eff du Soleil, revitalife^®^ (L’Oreal), nactosomes^®^ (Lancome), Formule liposome age^®^ (Payot), Future Perfect skin gel^®^ (Estée Launder), Symphatic 2000^®^ (Biopharm GmbH), Natipide II^®^ (Natterman), Aquasome LA^®^ (Nikko Chemical) and Eye perfecto^®^ (Avon).

The Nanobase^®^ cream patented by Yamanouchi appeared in Poland and was the first NLC-based product to be introduced to the market. This formulation has properties of good application, adherence and hydration of the skin. The bioactive molecules are dissolved in the aqueous phase of the cream. Dr. Rimpler GmbH, in Germany, has also launched a range of cosmetic products based on NLC, such as NanoRepair^®^Q10 and NanoVital^®^Q10. This range is able to incorporate a high concentration of co-enzyme Q10, the effect of which is intended for skin anti-aging. The first time that advertising for this range appeared was in October 2005 in Munich. More recently, the company Chemisches Laboratorium of Dr. Kurt Richter GmbH (Berlin in Germany) also launched in April 2006 its first NLC-containing cosmetic at the cosmetics fair in Barcelona [63].

Topical formulations aim to have local biological activity at a certain skin layer; if the drug reaches systemic circulation through this route, these formulations are called transdermal. Properties, such as molecular weight, pKa and partition coefficient govern the ability of a drug to permeate the skin layers. The hydrophobic composition of lipid carriers offers a suitable environment for the loading of lipophilic/hydrophobic bioactive ingredients and drugs while creating the opportunity to modify the release profile. Lipid carriers can act as permeation enhancers when able to reversibly disrupt stratum corneum to allow the drug entry, or when inducing an occlusive effect to increase skin hydration. The main limitation encountered with these systems is the toxicological risk encountered more by the use of surfactants and less by the type of lipid or mean particle size. Surface properties play an instrumental role in the interaction between nanoparticles and cells, and its surface electrical charge is therefore decisive in the initial contact with the negatively charged cell membrane. Additionally, in this context, cationic surfactants are, on the one hand, associated with a greater ability to insert into the cell membrane and create pores and defects in membrane integrity, but on the other hand, they sensitize the immune system. Indeed, cytotoxicity is dependent upon the concentration of cationic surfactant and can be reduced or non-existent with concentrations below 10 to 50 microg/mL. Most of the surfactants used in topical formulations are either neutral or negatively charged, which encounter no toxicological risk to cell lines [134].

## 7. Conclusions

With the increasing rise in exposure to solar radiation resulting in a continuous increase in the number of associated diseases, such as skin cancer, it makes the development of safer and more-effective photoprotectors a worldwide necessity and a constant challenge to the scientific community in general. The SLN and NLC used as adjuvants in photoprotection have proven to be advantageous when compared to the use of chemical and/or physical filters alone. The increase provided in photoprotection, photostability and the UV action spectrum, with the reduction in the concentration of filters and with the maintenance of the SPF maintaining the characteristics of the skin, are some direct benefits in the efficacy and safety of the SLN and NLC formulations. These advantages suggest that the introduction of these innovative lipid carriers in the market is only a matter of time, since the results are much more promising compared to the products currently commercialized. Lipid carriers have been developed in recent years for the treatment and prevention of wrinkles. However, most studies are aimed at improving formulations of clinically approved bioactive molecules. The use of nanotechnology aims at the future of the development of both cosmetics and cosmeceuticals, and other hygiene and beauty products. Although legislative norms are still unclear for the use of nanotechnology for topical use, companies already commercialize this technology. It should be noted that the vectors must be very well characterized in terms of their size, polydispersity and surface load. In addition, special attention should be paid to their interaction with biological systems, and further studies are needed to fully clarify the possible nanotoxicology. In the future, we believe that nanotechnology can revolutionize treatments related to skin aging, especially with the treatment and prevention of wrinkles, avoiding invasive surgical techniques.

## Figures and Tables

**Table 1:** Fitzpatrick scale for classification of skin types in relation to sensitivity to UV radiation.

Type	Description
I	Extremely clear skin, always burns, never tans
II	Fair skin, always burns, sometimes tans
III	Less clear skin, burns sometimes, tans always
IV	Light brown skin, rarely burns, always tans
V	Dark brown skin, never burns, always tans
VI	Black skin, never burns, always tans

## Data Availability

Not applicable.
